# Visualizing Biological Systems at the Molecular and Cellular Level at the Laboratory for BioMolecular Structure

**DOI:** 10.1063/4.0000888

**Published:** 2025-10-27

**Authors:** Liguo Wang, Guobin Hu, Jake Kaminsky, Qun Liu, Sean McSweeney

**Affiliations:** Brookhaven National Laboratory, Upton, NY, USA

## Abstract

In recent years, cryo-Electron Tomography (cryo-ET) has garnered increasing attention due to its unique capability for direct visualization of interactions between complexes in their cellular environment. It offers unparalleled insights into molecular organization, cellular structure, and cell physiology, making it a powerful tool for probing intricate details at the nanoscale within a cellular context.

The Laboratory for BioMolecular Structure (LBMS) at Brookhaven National Laboratory is a center for life science imaging that provides access to state-of-the-art cryo-electron microscopes (cryo-EM) and laboratory equipment for studying the building blocks of living organisms and their behavior. The focus is on complex interactions that define the function of entire biological systems, from molecules to organelles, cells, and multicellular organisms. With the establishment of LBMS, BNL provides peer-reviewed research access, support, and training for the use of cryo-EM.

To expand the cryo-ET capability at LBMS, we establish and operate a cryo-ET user program to support a broad range of projects. Three distinct routes will be offered based on the nature of the sample and the specific regions of interest (ROIs). With the development of the cryo-ET program, researchers can study cells/organelles and tissues. This bridges a critical imaging gap in the biomedical size spectrum, connecting studies of molecules at atomic resolution to cellular and tissue investigations.

